# Successful Treatment of Upper Limb Superficial Lymphatic Malformation With Topical Sirolimus

**DOI:** 10.1155/crdm/9574261

**Published:** 2025-12-26

**Authors:** Bahareh Abtahi-Naeini, Sara Adibfard, AliMohammad Sabzghabaee, Raha Moradi Gharibvand

**Affiliations:** ^1^ Pediatric Dermatology Division of Department of Pediatrics, Imam Hossein Children’s Hospital, Isfahan University of Medical Sciences, Isfahan, Iran, mui.ac.ir; ^2^ Skin Diseases and Leishmaniasis Research Center, Isfahan University of Medical Sciences, Isfahan, Iran, mui.ac.ir; ^3^ Student Research Committee, Isfahan University of Medical Sciences, Isfahan, Iran, mui.ac.ir; ^4^ Department of Clinical Pharmacy and Pharmacy Practice, School of Pharmacy and Pharmaceutical Science, Isfahan University of Medical Science, Isfahan, Iran, mui.ac.ir

## Abstract

Lymphatic malformation (LM) is a congenital anomaly of the lymphatic system that can affect various anatomical sites, most commonly skin and subcutaneous tissues. Treatments that were historically used, including surgery, laser therapy (pulsed dye laser [PDL] and CO2 laser), and sclerotherapy, can be associated with complications such as bleeding and lesion recurrence. Oral sirolimus is effective in treating LMs but can be associated with systemic side effects, including immunosuppression and metabolic disturbances. Targeting the mTOR pathway, topical sirolimus effectively treats superficial LMs with minimal adverse effects compared to systemic administration. Several studies have reported that topical sirolimus shows comparable outcomes with minimal side effects. We present the case of a 19‐year‐old woman with bleeding from a superficial LM on the lateral aspect of her upper limb. Previous treatments, including carbon dioxide laser therapy and PDL, were ineffective. Within 3 months after initiating 0.1% topical sirolimus application twice daily, a noticeable reduction in lesion size and bleeding was observed, with no adverse effect.

## 1. Introduction

Lymphatic malformations (LMs) are benign congenital malformations of the lymphatic system, with an incidence of 1 in 2000–4000 live births [1]. Classified as macrocystic (> 1 cm), microcystic (< 1 cm), or mixed, LMs are typically diagnosed in early childhood via clinical history and physical examination [2]. Commonly affecting the head and neck, they cause symptoms ranging from localized swelling to life‐threatening complications [3]. Complications, including infection, bleeding, and disfigurement, necessitate effective treatments. While traditional options include surgery, sclerotherapy, and laser therapy, many LMs are now treated medically with sirolimus and other targeted agents, reflecting a major shift in management over the past decade. [4, 5]. However, these treatments often cause pain, scarring, and incomplete resolution. Sirolimus, a macrolide with immunosuppressive properties, shows promise for treating LMs. While oral sirolimus is effective, it brings risk of systemic side effects, and topical sirolimus has demonstrated efficacy in cutaneous vascular anomalies, including LMs, with minimal adverse effects [6, 7]. This case report evaluates the use of topical sirolimus for the management of a cutaneous microcystic lymphatic malformation (cmLM). The noteworthy aspect of the present case, which distinguishes it from previously published reports, is the remarkable clinical response achieved with a low‐dose concentration of topical sirolimus in a patient who had failed to respond to conventional therapies.

## 2. Case Presentation

A 19‐year‐old female patient presented with a vascular plaque and multiple hemorrhagic papules on the lateral aspect of her left arm. The patient reported that the lesions, present since birth, had progressively worsened over time, causing significant distress. In the preceding months, the patient experienced recurrent bleeding episodes triggered by minor trauma (Figure 1(a)).

Figure 1(a) Clinical presentation of the superficial lymphatic malformation. Multiple hemorrhagic papules and vesicles are visible, some with crusting due to recurrent bleeding. (b) Same lesion after 6 months of twice‐daily application of 0.1% topical sirolimus. There is a marked reduction in the size and number of hemorrhagic vesicles, decreased bleeding tendency, and improvement in the overall appearance of the lesion, with no sign of local irritation or adverse effect.

**Figure:**
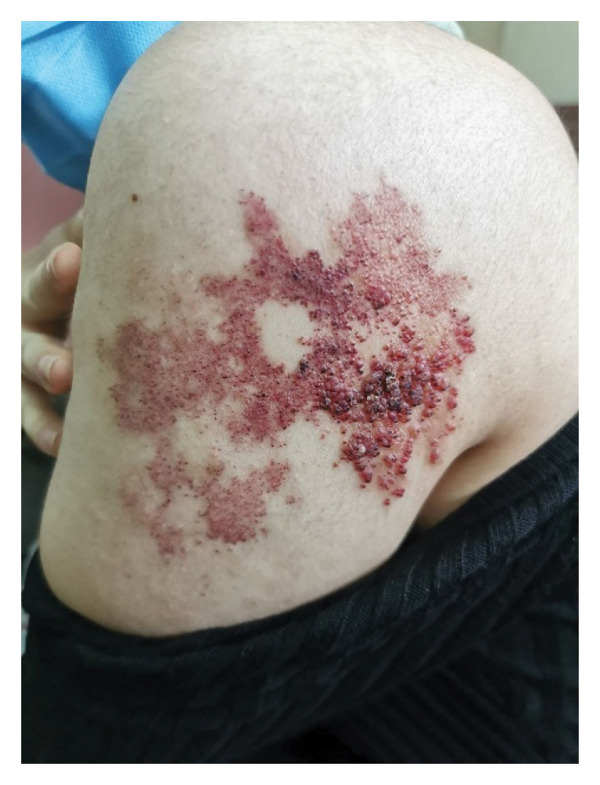
(a)

**Figure:**
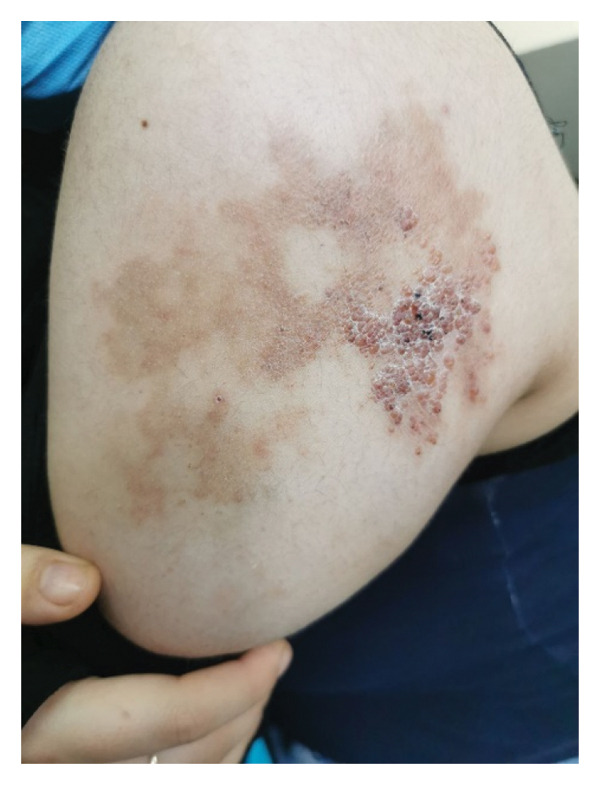
(b)

Initially, one session of pulsed dye laser (PDL) therapy was performed but discontinued due to significant bleeding. Thereafter, carbon dioxide laser therapy was recommended, but the patient was unable to pursue further treatment. Consequently, 0.1% topical sirolimus was prescribed and applied twice daily as a thin layer to the lesion surfaces. The formulation was prepared by crushing twenty 1‐mg sirolimus tablets into a fine powder, sieving it through a fine mesh, and mixing it with a suitable base. Five mL of ethanol was added to the powder to form a paste, which was then levigated with a suitable base to yield 20 mL of a 0.1% sirolimus semisolid formulation. Written informed consent was obtained from the patient for the publication of clinical photographs and treatment details before initiating therapy.

## 3. Discussion

Sirolimus, a macrolide derived from *Streptomyces hygroscopicus*, inhibits the mammalian target of the rapamycin (mTOR) pathway. Somatic mutations in genes such as PIK3CA and AKT are implicated in the mTOR pathway; the RAS/Raf/MAPK pathway has distinct cross‐talk. Sirolimus binds to FKBP12, an immunophilin, forming the FKBP12‐sirolimus complex. This complex inhibits mTORC1 by binding to its FKBP12‐rapamycin binding domain (FRB), preventing autophosphorylation. This inhibition prevents substrate binding, blocking G1‐to‐S phase progression in the cell cycle. Sirolimus also suppresses hypoxia‐inducible factor 1*α* (HIF‐1*α*) expression, reducing hypoxia‐induced angiogenesis. Sirolimus inhibits the PI3K/Akt pathway, which mediates vascular endothelial growth factor (VEGF) signaling, thereby reducing endothelial differentiation and vascular regeneration [2–4].

Ivars et al. reported a case of lymphorrhea and lymphangitis treated with 0.8% topical sirolimus, with no adverse effects observed [3]. Çalıkan et al. also used topical sirolimus in the concentration of 0.2% to treat. However, in this case, an eczematous reaction on the treatment site is shown as an adverse effect [6]. Montero et al. successfully treated two cases of cmLM with 1% sirolimus ointment [2]. One case experienced increased swelling and discomfort in the right buttock after treatment. Leducq et al. also use sirolimus 1% ointment for a patient with cmLM. It was treated efficiently, but slight tingling was observed during the treatment [7]. Badia et al. reported a case series of 23 patients with vascular anomalies, mostly LM with cutaneous blebs, who were treated with 1% topical sirolimus. The majority had previously received local intervention, and treatment was applied twice daily for a median of 622 days. Clinical improvement was observed in 86% of the patients, with the highest response in those with lymphatic bleeding (89%). No major systemic side effects were reported, and only one patient discontinued due to local irritation [4]. Yonekura et al. used 0.1% topical sirolimus, with positive outcomes and no adverse effects [5]. In Table 1, we summarize these limited studies evaluating topical sirolimus as a therapeutic approach for lymphatic disorders. These findings suggest that topical sirolimus may represent a promising and practical treatment option for LM. The relatively short follow‐up period limits our ability to assess the long‐term efficacy, recurrence rate, and potential delayed adverse effects of topical sirolimus therapy. Nevertheless, the favorable clinical response observed with an exceptionally low concentration (0.1%) of topical sirolimus highlights the need for further prospective studies involving larger cohorts to validate its therapeutic role in LMs.

**Table 1:** Topical sirolimus treatment for patients with superficial lymphatic malformation.

First author/year	Age (year)/gender	Localization	Previous treatment	Treatment protocol	Outcomes	Adverse effects
Ivars and Redondo [3]/2016	In his 20s/M	Scrotum and the penis root	Curettage Intralesional bleomycin sclerotherapy (one session with recurrent and new episodes of lymphorrhea)	0.8% topical sirolimus (daily for 3 months)	Lymphorrhea practically disappeared, and the size of the lesion reduced	No adverse effect

Çalıkan et al. [6]/2017	8/F	Left trunk	Surgical operation (recurrence of lesions happened)	0.2% topical sirolimus (once a day for 19 weeks)	The lesions were almost cleared of irritation	Eczematous reaction on the treatment site

García‐Montero et al. [2]/2017	5/M	The internal quadrant of the left buttock	Surgery (an LM developed above the scar). Laser multiplex treatment IBI	Sirolimus 1% ointment (twice a day for the first month and once a day for another 5 months)	A significant reduction in size, number of vesicles, and exudation, with no new episodes of superinfection or bleeding	No adverse effect

García‐Montero et al. [8]/2017	13/F	Right buttock	Cryotherapy (the treatment provoked intense local irritation)	Sirolimus 1% ointment (twice a day for the first month and once a day for 3 months)	General flattening of the lesions with no new episodes of bleeding, malodorous exudate, and fever spikes. A significant reduction in pain	Increased swelling and discomfort in the right buttock

Leducq et al. [7]/2019	17/M	Gluteal area	Sclerotherapy. CO2 laser. Retinoid therapy	Sirolimus 1% ointment (twice a day for the first month and once a day for 5 months)	Reduction of hyperkeratosis and the number of vesicles, thickness, oozing, and bleeding	Slight tingling during the first month

Badia et al. [4]/2019	23 patients, ranging from 4 to 27/15 F and 8 M	LM, capillary‐venous‐lymphatic malformation (CLVM) [5], venous‐lymphatic malformation (LVM) [3], capillary malformation (CM) [2]	82% had prior surgery, laser, or sclerotherapy; 6 patients on systemic sirolimus	Topical 1% sirolimus, twice daily; duration 109–1424 days	86% overall improvement; 89% of LM/mixed with blebs improved; ¾ without blebs improved	No major systemic side effects: 1 patient stopped due to local pruritus and burning

Yonekura et al. [5]/2022	17/M	Urethra	Surgery (the lesions relapsed 4 months later). Cryotherapy (the lesions did not disappear)	Sirolimus (0.75 mg/mL) in lotion form (twice a day and then once daily for 3 months)	Reduction of LM lesions. Recurrence of lesions	No adverse effect

Our case	19/F	Arm	PDL. CO2 laser	Topical sirolimus at 0.1% (twice a day)	Lesions were significantly improved, with no new episodes of bleeding	No adverse effect

*Note:* M: male; F: female; CO2 laser: carbon dioxide laser; mg: milligram; ml: milliliter; CLVM: capillary‐venous‐lymphatic malformation, LVM: venous‐lymphatic malformation.

Abbreviations: IBI = intralesional bleomycin injection, LM = lymphatic malformation, PDL = pulsed dye laser.

## Disclosure

We affirm that no authors are employed by a government agency primarily dedicated to activities other than research and/or education. Furthermore, none of the authors serves as an official representative of the government. All authors participated in the critical review of the manuscript for important intellectual content, approved the final version for submission, and agreed to be accountable for all aspects of the work.

## Conflicts of Interest

The authors declare no conflicts of interest.

## Author Contributions

Bahareh Abtahi‐Naeini: conceptualization, methodology, writing–original draft, and supervision.

AliMohammad Sabzghabaee: writing–review and editing, validation, and supervision.

Sara Adibfard: data curation, literature review, and writing–draft preparation.

Raha Moradi Gharibvand: data curation, visualization, and writing–draft preparation.

## Funding

The authors do not have any financial disclosures. Furthermore, they received no financial support for this article’s research, drafting, or publishing.

## Data Availability

The data supporting the findings of this study are included within the article and its supporting information. Raw data are available upon reasonable request from the corresponding author, subject to ethical and legal restrictions.
